# Cloning and Expression Characteristics of the Pig *Stra8* Gene

**DOI:** 10.3390/ijms150712480

**Published:** 2014-07-15

**Authors:** Xiaoyan Wang, Tingfeng Chen, Chengyi Song, Bo Gao, Yani Zhang

**Affiliations:** College of Animal Science & Technology, Yangzhou University, Yangzhou 225009, China; E-Mails: ctf20070702664@126.com (T.C.); cysong@yzu.edu.cn (C.S.); bgao@yzu.edu.cn (B.G.); ynzhang@yzu.edu.cn (Y.Z.)

**Keywords:** *Stra8*, Meishan pig, expression

## Abstract

*Stra8* (Stimulated by Retinoic Acid 8) is considered a meiotic gatekeeper gene. Using reverse transcriptase PCR and rapid amplification of cDNA ends (RACE), the complete sequence of the pig *Stra8* gene was cloned. Bioinformatics analyses of this sequence were performed. Using semi-quantitative methods, the expression characteristics of *Stra8* in Testis, cauda epididymis, body epididymis, caput epididymis, seminal vesicles, prostate gland, Cowper’s gland, heart, liver, spleen, lung, kidney, stomach, hypothalamus, pituitary gland, cerebrum, cerebellum, and hippocampus of adult Meishan boar and sow tissues were examined. The expression pattern in the testis of 2-, 30-, 60-, 90-, and 150-day old Meishan boars were analyzed using real-time PCR. We constructed a eukaryotic expression vector for the *Stra8* gene and used it to transfect NIH-3T3 cells and third generation pig spermatogonial stem cells (SSCs) cultured *in vitro*. Testes weight and sperm count in the cauda epididymis were evaluated at various time points. The results showed that the length of the pig *Stra8* gene cDNA was 1444 bp encoding 366 amino acids with one typical helix-loop-helix (HLH) domain. It is testes-specific expression. Expression was first detected in boar testis starting at day 2, and its expression significantly (*p* < 0.05) increased with age and body weight. When NIH-3T3 cells and pig SSCs were transfected with the eukaryotic expression vector EGFP (enhanced green fluorescent protein)-N1-p*Stra8*, it was expressed in the cytoplasm of NIH-3T3 cells. However, in SSCs, *Stra8* was expressed predominantly in cytoplasm and few in nucleus. Our data suggest that perhaps *Stra8* acts as a transcription factor to initiate meiosis in young boar.

## 1. Introduction

In mammals, haploid gametes must undergo meiosis from germ cells whether they are male or female. The timing of meiosis entry differs significantly between male and female germ cells. At nearly 13.5 days post-coitum (dpc), female primordial germ cells (PGCs) initiate meiotic process and then arrest at the dictyate stage, whereas male PGCs arrest at the G0/G1 stage of the cell cycle in the mitotic process. After birth, male PGCs resume mitotic proliferation and subsequently enter meiosis during puberty [1,2,3,4]. Retinoic acid (RA) has been proven to induce meiotic initiation for PGCs [5,6]. 

Vitamin A deficiency results in meiotic failure and the accumulation of undifferentiated spermatogonia in prepubertal mouse testis [7]. RA is an active metabolite of Vitamin A and a vital signaling molecule for many physiological processes [8]. The effects of RA were mediated by retinoic acid receptor A (RARA) through activating *Mafb* expression to germ cell differentiation [9]. It can induce expression of *Stra8* (Stimulated by Retinoic Acid 8) leading to the onset of meiosis in both male and female gonads. *Stra8* was first identified in 1995 as one of the RA-responsive genes in P19 embryonic carcinoma cells [10,11]. In female PGCs, RA can induce *Stra8* gene expression at 12.5 dpc and lead to meiotic initiation. However, in male fetal testes, CYP26B1, produced by pre-Sertoli cells, can metabolize RA to prevent male PGCs from entering meiosis. Therefore, male PGCs maintain mitosis and after birth, they can enter into meiosis with expression of *Stra8* [6,12,13]. Exogenous RA can induce or enhance *Stra8* gene expression in SSCs cultured *in vitro* [14,15,16,17]. 

Knockout of the *Stra8* gene blocks entry into meiosis in both embryonic ovaries and pubertal testes [18,19]. The early mitotic development of germ cells appears normal in juvenile C57BL/6 male mice. However, these cells cannot finish meiosis and have no leptotene, zygotene, or pachytene spermatocytes cells in testes. Therefore, *Stra8* is necessary for processes including chromosomal cohesion, synaptonemal complex formation, and recombination and acted as a meiotic gatekeeper gene [19,20]. Gene knockdown and transgenic models of some genes involved in RA synthesis and metabolism also prove that *Stra8* gene expression is induced by RA and is important for the onset of meiosis [7,21]. The genetic variant and expression change of *Stra8* showed higher risk of spermatogenic impairment in the groups of infertility, which implies Stra8 is critical in spermatogenesis [22,23]. 

The Meishan pig is notable for its high prolificacy and early sexual maturity [24]. In male Meishan pigs, the onset of puberty (spermatogenesis) starts at a much younger age (56–84 days) than that of other conventional pigs (120–180 days) [25]. Spermatogenesis begins from PGC meiosis after birth with onset of boar puberty. *Stra8* gene is considered a meiotic gatekeeper gene. In order to better understand the characteristics of this gene, we cloned it from Meishan boar testes and studied its spatio-temporal expression pattern. 

## 2. Results and Discussion

### 2.1. Cloning of Pig Stra8

PCR amplification using the Meishan boar testes cDNA yielded 1108 bp middle sequence, 254 bp 5' end, and 157 bp 3' end. The fragments were cloned and sequenced. The full-length *Stra8* gene cDNA was 1444 bp, assembled using the three sequences. Basic Local Alignment Search Tool (BLAST) searches showed that it had the highest sequence identity to mammalian *Stra8*. The pig *Stra8* cDNA was submitted to the NCBI GenBank (accession number JQ965783). 

**Figure 1:**
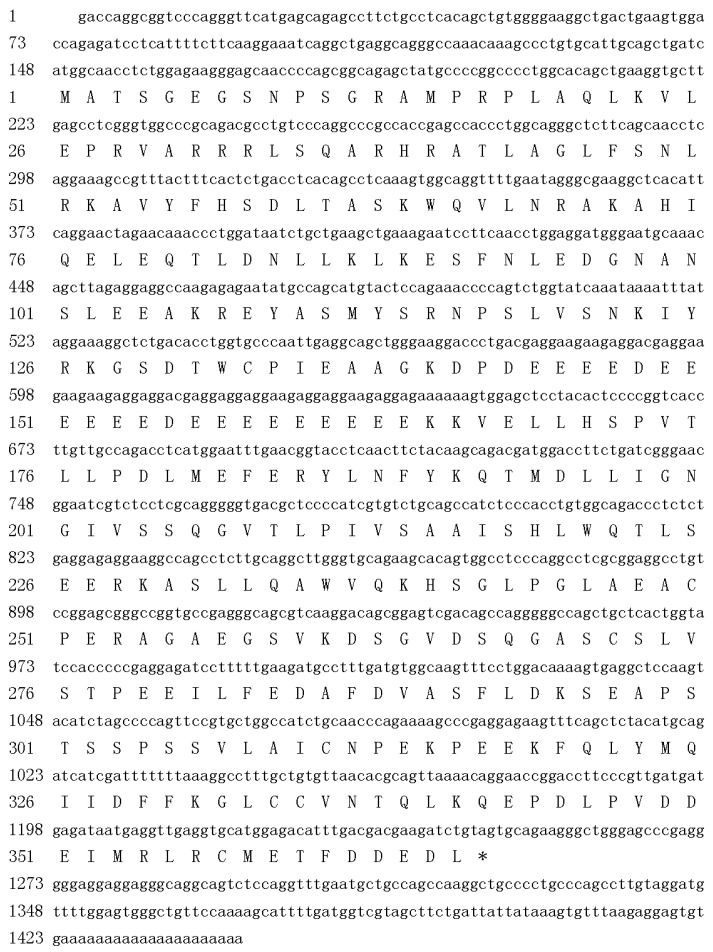
Nucleotide and amino acid sequence of pig *Stra8.* Stop codon is shown as “*”.

Using Open Reading Frame Finder (ORF Finder), the 1444-bp pig *Stra8* cDNA contains 147 bp 5' end, 1101 bp ORF, and 196 bp 3' end. It encodes a protein of 366 amino acids with a molecular mass of 41.04 kDa. The sequence can be found with a polyadenylation signal sequence (TATAAA) and a polyadenylation tail (Figure 1).

**Figure 2:**
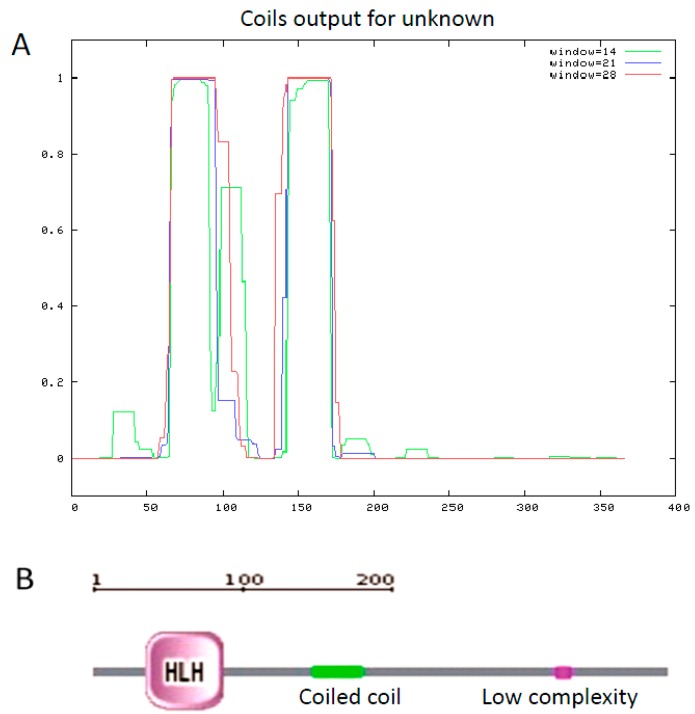
Analysis of the functional domains of the *Stra8* amino acid sequence. (**A**) using COILS program; (**B**) using SMART program.

Among the secondary structure elements, alpha helices were predominant, followed by loops. Stra8 is not a secreted protein, nuclear protein, or DNA binding protein. Two coiled-coil regions range from amino acids 62–95 and 143–170 based on the predicted amino acid sequence by COILS (SIB, Lausanne, Switzerland). Based on predictions by SMART (EMBL, Heidelberg, Germany), amino acids 34–84 of pig STRA8 is a helix-loop-helix (HLH) structure and amino acids 140–172 is a coiled-coil domain (Figure 2). Transmembrane domains and signal peptides were not found in the polypeptide chain.

### 2.2. Multiple Sequence Alignments and Phylogenetic Relationships

**Figure 3:**
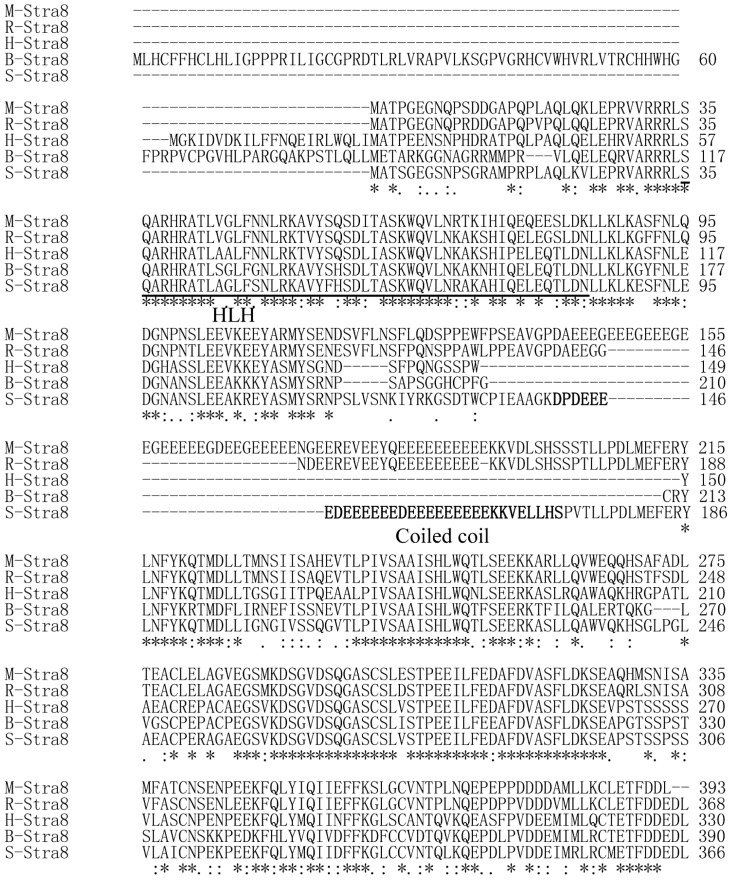
Amino acid sequence alignment of STRA8. M-STRA8: mouse STRA8; R-STRA8: rat STRA8; H-STRA8: human STRA8; B-Stra8: bovine STRA8; S-STRA8: pig Stra8. HLH: helix-loop-helix. “*” the amino acid is identical in this position; “:” the amino acid in this position is conserved; “.” the amino acid in this position is semi-conserved. Pig HLH structure is underlined and Coiled coil is bold.

**Figure 4:**
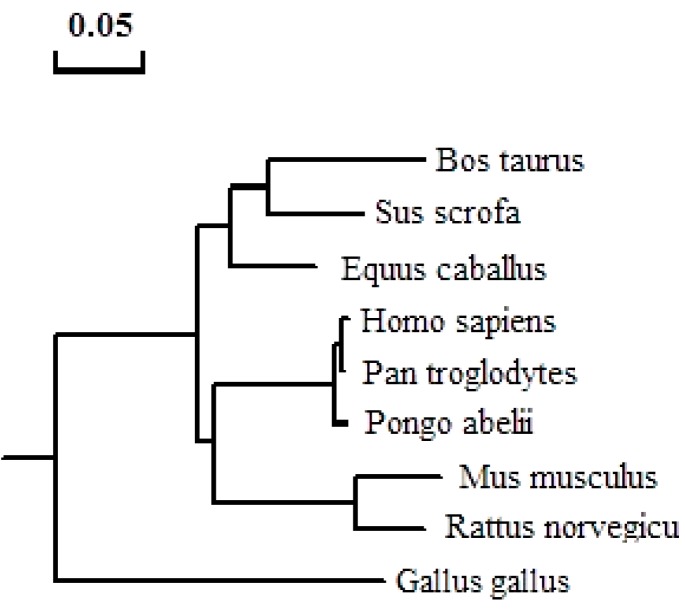
Phylogenetic analyses of the *Stra8* gene.

The pig STRA8 amino acid sequence shares the highest similarity (78.4%) with mouse, and has the lowest homology (45.4%) with chicken (Table 1). Using the Clustal W program to analyze Stra8 amino acid homology among different species, it was highly conserved in the predicted HLH structure and 3' end between pig and human, rat, mouse, and cattle. There is a glutamic acid rich domain in the middle of the pig STRA8 amino acid sequence that exists only in rat and mouse STRA8 (Figure 3). A phylogenetic tree was constructed using DNAMAN to analyze phylogenetic relationships. Pig STRA8 clusters with domestic breeds (cattle and horse), but not with humans and other primates such as chimpanzee and Sumatran orangutan. Pig STRA8 was least related to chicken (Figure 4).

**Table 1:** Homology analyses of STRA8 amino acid sequence.

Species	Similarity	GenBank Accession Number
Homo sapiens	66.5%	NM_182489.1
Rattus norvegicus	72.1%	XM_575429.2
Mus musculus	78.4%	NM_009292.1
Bos taurus	57.8%	XM_001253649.1
Canis lupus familiaris	66.4%	XM_847727.2
Gallus gallus	45.4%	XM_416179.3
Equus caballus	75.0%	XM_001914766.1
Pongo abelii	66.8%	XM_002818472.1
Pan troglodytes	66.6%	XM_001144872.2

### 2.3. Stra8 mRNA Distribution

**Figure 5:**
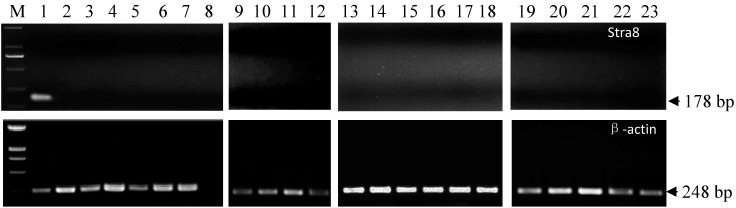
Expression of *Stra8* mRNA in reproductive tissues from Meishan boar and sow. **M**: DL2000; **1**: Testis; **2**: Caput epididymis; **3**: Body epididymis; **4**: Cauda epididymis; **5**: Seminal vesicle; **6**: Prostate; **7**: Balbocavernous glands; **8**: Negative control; **9**: Ovary; **10**: Uterus corners; **11**: Uterus; **12**: Oviduct; **13**: Heart; **14**: Liver; **15**: Spleen; **16**: Lung; **17**: Kidney; **18**: Stomach; **19**: Hypothalamus; **20**: Pituitary gland; **21**: Cerebrum; **22**: Cerebellum; **23**: Hippocampus.

Semi-quantitative RT-PCR was performed to analyze *Stra8* gene expression patterns in testis, cauda epididymis, body epididymis, caput epididymis, seminal vesicles, prostate gland, Cowper’s gland, heart, liver, spleen, lung, kidney, stomach, hypothalamus, pituitary gland, cerebrum, cerebellum, and hippocampus of adult boars and sows. *Stra8* expression was exclusively expressed in the testes (Figure 5).

### 2.4. Developmental Expression Pattern of Stra8, Testes Weight, and Sperm Count

**Figure 6:**
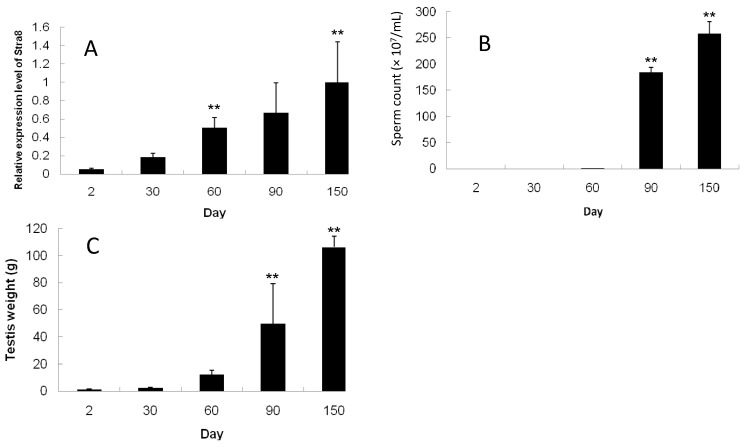
Expression of *Stra8* mRNA in testis, the sperm count in cauda epidydimis, and testes weight of Meishan boars at various time points. (**A**) Expression of *Stra8* mRNA; (**B**) Sperm count; and (**C**) Testes weight. Significant increases are denoted with “**” (compared with their previous stage, *p* < 0.01).

Using real-time quantitative PCR, *Stra8* mRNA expression was evaluated in pig testes at different ages. β-actin was used as an internal reference and *Stra8* expression at 150 days were used as a control. Testes *Stra8* mRNA was expressed from 2 days post partum and increased significantly from day 60 until it peaked at day 150 (*p* < 0.01; Figure 6A). Sperm with complete morphological characteristics were observed until day 60 in cauda epididymis (Figure 6B). The sperm count increased dramatically on days 90 and 150 (*p* < 0.01). There were no differences among 2-, 30-, and 60-day testis weight. However, after day 60, testis weight increased significantly (*p* < 0.01) (Figure 6C). It is therefore possible that the high levels of *Stra8* mRNA expression on day 60 were in preparation for the subsequent production of large numbers of sperm and sex maturity.

### 2.5. Subcellular Localization of Pig Stra8 in NIH-3T3 Cells and Pig SSCs (Spermatogonial Stem Cells)

**Figure 7:**
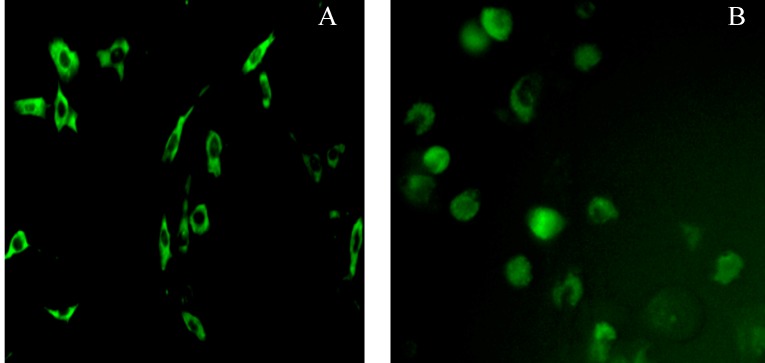
NIH-3T3 cells and pig SSCs (spermatogonial stem cells) transiently expressing EGFP-N1-*Stra8*. (**A**) NIH-3T3 cells transfected with pEGFP-N1-*Stra8*; (**B**) Pig SSCs transfected with pEGFP-N1-*Stra8* (×200).

*Stra8* ORF fragment were inserted into EGFP-N1 vector. After double enzyme digestion and sequencing, the correctly inserted *Stra8* ORF fragment was named pEGFP-N1-*Stra8*. In order to investigate the subcellular location and function of pig *Stra8* in meiosis, the protein was equipped with a GFP tag fused to its *C*-terminus and transiently transfected into NIH-3T3 cells and pig SSCs. Subsequent analyses using fluorescence microscopy revealed that pEGFP-N1-*Stra8* was exclusively localized to the cytoplasm in 3T3 cells (Figure 7A) and predominantly localized to the cytoplasm in pig SSCs (Figure 7B). 

### 2.6. Discussion

The *Stra8* gene is one of 50 RA-responsive genes isolated from a P19 pluripotent embryonal carcinoma (EC) cell line treated with RA. The expression of *Stra8* in spermatogonia coincides with the transition from undifferentiated type A spermatogonia to differentiated type A1 spermatogonia. *Stra8* cDNA (1455 bp) is located on mouse chromosome 6 and encodes a putative 393 amino acid protein containing a glutamic acid-rich domain [9]. Human *Stra8* mRNA is 993 bp and encodes a 330 amino acid protein that maps to chromosome 7 [26]. In this study, based on GenBank Expressed Sequence Tags (ESTs), we used RT-PCR, 5'-RACE, and 3'-RACE to obtain a 1444 bp pig *Stra8* cDNA that encodes a putative 367 amino acid protein. Similar to the mouse amino acid sequence, the putative pig *Stra8* amino acid sequence has a glutamic acid-rich domain. This domain can form coiled-coil structures as shown using COILS and SMART. Using Protscale, the domain is highly hydrophilic, perhaps to improve the stability of the coiled-coil domain in the aqueous cytoplasmic environment [27]. Glutamic acid-rich domains were found in pig, mouse, and rat *Stra8* amino acid sequences, but not in human and cattle. The function of this domain requires further study. The *Stra8* HLH structure identified by SMART had relatively high conservation between pig and other species. The HLH motif comprises two amphipathic α-helices of 15–16 residues separated by a loop and is responsible for dimerization of a class of transcription factors called HLH proteins [28]. Therefore, it is possible that *Stra8* acts as a transcription factor during the onset of spermatogenesis.

Oulad-Abdelghani *et al.* (1996) [9] showed that *Stra8* expression is restricted to adult mouse testes. After treatment with RA, P19 and F9 cells, as well as SSCs, began to express *Stra8* mRNA within 24 h. These results show that RA can induce some cell lines that are not germ cells to express *Stra8*. Human *Stra8* is also expressed selectively in testes [26]. In this study, we found that among 19 Meishan boar tissues and four Meishan sow reproductive tissues, *Stra8* is expressed only in adult testes. Mouse *Stra8* could be detected in germ cell as early as five days postpartum and its expression must coincide with the initiation of meiosis in postnatal testes [29]. When we evaluated the developmental expression pattern in postnatal Meishan boar testes, *Stra8* mRNA can be detected at two days postpartum. However, its expression increased significantly with the age and bodyweight. Meishan, a Chinese native breed famous for its prolificacy, undergoes pubertal development at a younger age (56–84 days) than conventional breeds (120–180 days) for boar [25]. Spermatocytes and spermatids increased significantly in seminiferous tubules of Erhualian boar from day 60 and sperm appears [30]. Based on sperm counts in cauda epidydimis from Meishan boar, sperm with recognizable characteristics emerged at day 60 in this study. The spermatogenic cycle ranges from the period of spermatogonia launch meiosis until sperm finally develop with integrated heads and tails *in vivo*. The pig spermatogenic cycle is 42 days. We conclude that spermatogonia in Meishan boar testis must initiate meiosis before day 18 postpartum. *Stra8*, a meiotic gatekeeper gene, is expressed in premeiotic germ cells and can be detected at two days postpartum in Meishan boar. Whether *Stra8* levels could impact the onset of meiosis 2–18 days after birth needs be studied further. Sperm can be first found in semen at different time points in Erhualian boar (60–75 days) and Jiling black pig (a breed inbred with Chinese native pigs and western-introduced pigs, 134–143 days) [31]. These time points haveeconomic importance to boar breeding, and whether they are attributed to the start of meiosis initiation or another mechanism is unclear. Single nucleotide changes in *Stra8* have been detected that are absent in men with normozoospermia [32]. Because subsequent functional analyses of the changes were likely not the cause of infertility, it can be concluded that mutations are rarely detected in men with fertility problems. Therefore, further research is necessary to determine the impact of genetic causes on boar meiosis initiation. 

In order to study the mechanism of *Stra8* gene function in spermatogenesis *in vitro*, we constructed EGFP-recombinant expression vectors and transfected them into NIH-3T3 cells. The fusion protein was only localized in the cytoplasm. This result is similar to Miyamoto *et al.* (2002) [26] and Oulad-Abdelghani *et al.* (1996) [9] because it is not localized in other cytoskeletal structures. However, when the plasmid was transfected into pig SSCs, fluorescence was observed in the cytoplasm as well as the nucleus. *Stra8* has previously been found in the nucleus in P19 cells and F9 cells [33]. In this study, we found pig *Stra8* has a HLH structure based on sequence analyses and is located in the nucleus of some SSCs. Therefore, it can be predicted that *Stra8* may be act as a transcription factor to initiate pig SSCs meiosis. However, because the transfection efficiency is relatively low, we need to improve this technical issue and study *Stra8* localization further.

## 3. Experimental Section

### 3.1. Animals and Samples

Meishan boars and sows were obtained from six multiparous dams and were housed together prior to sacrifice. All piglets were weaned at 28 days and fed standard pelleted rations. The animal use protocol was approved in accordance with the University Council on Animal Care guidelines.

Testis, cauda epididymis, body epididymis, caput epididymis, seminal vesicles, prostate gland, Cowper’s gland, heart, liver, spleen, lung, kidney, stomach, muscle, hypothalamus, pituitary gland, cerebrum, cerebellum, and hippocampus samples from six 150-day-old boars as well as ovary, uterus corners, cervix, and oviduct samples from three 150-day-old sows, were rapidly dissected after sacrifice and frozen in liquid nitrogen for tissue specific detection. Each right testis from 2- (*n* = 5), 30- (*n* = 4), 60- (*n* = 3), 90- (*n* = 3), and 150- (*n* = 6) day-old boars were obtained and frozen in liquid nitrogen for detection of developmental gene expression. Left testes were weighed and the left cauda epididymis was used to count sperm number [34].

### 3.2. Total RNA Isolation and cDNA Synthesis

Total RNA was extracted from frozen tissues using Trizol reagent (Invitrogen, Carlsbad, CA, USA) according to the manufacturer’s instructions. RNA concentrations were quantified spectrophotometrically at 260 nm. RNA (1 μg) was used to synthesize cDNA using the AM-MLV Reverse Transcriptase kit (Invitrogen) in a reaction volume of 50 µL. First strand cDNA (1 μL) was used for PCR reactions.

### 3.3. Cloning of Pig Stra8 Gene

The Human stra8 CDS was used as a bait to obtain highly similar pig ESTs from dbESTs (National Center for Biotechnology Information, NCBI, Bethesda, MD, USA) using BLASTn. Primers were designed using primer 3.0 online [35] and used to confirm the sequence assembled by these dbESTs. The primers used were 5'-ATGGCAACCTCTGGAGAAGG-3' (forward) and 5'-CAGATCTTCGTCGTCAAATG-3' (reverse). PCR amplification in Meishan boar testis was performed using rTaq polymerase (Takara, Dalian, China) in a 25 μL reaction mixture consisting of an initial 5 min denaturation step at 95 °C, 35 cycles of 95 °C for 45 s, 56 °C for 45 s, 72 °C for 1 min, and a final extension for 10 min at 72 °C. The amplified DNA fragments were subsequently cloned into pGEM-T Easy (Promega, Madison, WI, USA) and evaluated by Shanghai GeneCore BioTechnologies Co., Ltd. (Shanghai, China).

### 3.4. 5'-RACE (Rapid Amplification of cDNA Ends) and 3'-RACE

Primers for 5'-RACE and 3'-RACE were designed based on the sequences obtained above. 5'-RACE was performed according to a previously described method [36,37]. Specific cDNAs were reverse transcribed in boar testes using the gene specific primer P51 (5'-CCTGGGGTTTCTGGAGTACA-3'). The first PCR cycle used special primer P52 (5'-TCTTGGCCTCCTCTAAGCTG-3') and the adaptor primers Q_O_ and Q_T_. Diluted PCR products were used in the second cycle with special primer P53 (5'-CTCAAGCACCTTCAGCTGTG-3') and adaptor primer Q_I_ to amplify the 5'-end fragment. PCR programs for RACE reactions were similar to those described above. 

3'-RACE was performed as described [36,38]. Testes cDNA was reverse transcribed using adaptor primer Q_T_. The first cycle PCR used special primer P31 (5'-GTGCATGGAGACATTTGACG-3') and adaptor primer Q_O_. Diluted PCR products were used in the second cycle with special primer P32 (5'-GGCAGTCTCCAGGTTTGAAT-3') and adaptor primer Q_I_ to amplify the 3'-end fragment. The second PCR products from 5'-RACE and 3'-RACE were cloned and sequenced. The full-length *Stra8* gene was assembled using these fragments and Megalign of DNASTAR (DNASTAR, Inc., Madison, WI, USA) and submitted to NCBI.

### 3.5. Sequence Analyses

The protein secondary structure using Predictprotein [39]. Coiled-coiled structures were predicted using COILS [40]. Conserved protein domains were identified using the SMART databases [41]. Amino acid similarities were performed using the Multiple Sequence Alignment Program Clustal W of the EMBL-EBI database [42] according to the default alignment parameters. Phylogenetic analyses of deduced amino acid alignments were performed by DNAMAN (LynnonBiosoft, Vaudreuil, QC, Canada) using the neighbor-joining method.

### 3.6. Semi-Quantitative RT-PCR

*Stra8* gene expression was investigated in different tissues from Meishan boars and sows using semi-quantitative RT-PCR. The forward primer 5'-ACCTCACAGCCTCAAAGTGG-3' and reverse primer 5'-CCTGGGGTTTCTGGAGTACA-3' were designed to detect tissue expression characteristics based on the sequences obtained above. RNA isolation, reverse transcription, and PCR programs were performed as described above. The β-actin gene (forward primer 5'-GCTGTATTCCCCTCCATCGT-3' and reverse primer 5'-ACGGTTGGCCTTAGGGTTCA-3') was used as a positive control. The number of PCR cycles (ranging from 25 to 35 cycles) was optimized for each gene. All experiments were repeated three times. PCR products were separated on ethidium bromide-stained 1.5% agarose gels.

### 3.7. Quantitative Real-Time RT-PCR Assays

Meishan boar testes tissue (2-, 30-, 60-, 90-, and 150-day-old) was used to evaluate the developmental expression of *Stra8*. Real-time PCR was performed using the 7500 System (ABI, Carlsbad, CA, USA) in a total volume of 20 μL containing SYBR mix (10 μL; Takara), primers (4 ng), and cDNA sample (50 ng) according to the manufacturer’s instructions. Pig β-actin was used as an internal reference to normalize relative gene expression.

*Stra8* gene expression was measured using the 2^−ΔΔ*C*t^ method. According to the method, β-actin *C*t values were used as the internal reference and *Stra8* *C*t values at 150 days were used as control. ΔΔ*C*t = (*C*t_target genes_ − *C*t_β-Actin_) at 2, 30, 60, and 90 days − (*C*t_target genes_ − *C*t_β-Actin_) at 150 days. All PCR products were run on ethidium bromide-stained agarose gels and confirmed using melting curve analyses to assess product quality.

### 3.8. Construction of Expression Vectors

Pig *Stra8* cDNA was PCR-amplified from an adult boar testicular cDNA library using the method described above and EX-Taq polymerase (Takara). The PCR primers used were 5'-CCAAGCTTATGGCAACCTCTGGAGAAGG-3' (forward) and 5'-CGCGGATCCCAGATCTTCGTCGTCAAATG-3' (reverse) (Hind III and BamHI restriction sites underlined), which were constructed based on the sequence obtained above. PCR amplification consisted of an initial 5-min denaturation step at 96 °C, followed by 35 denaturation cycles at 96 °C for 30 s, annealing at 65 °C for 30 s and extension at 72 °C for 1 min, and a final 10-min extension. The resulting PCR products and EGFP-N1 vector (Clontech, Mountain View, CA, USA) were double digested with Hind III and BamHI restriction enzymes and ligated at 16 °C for 16 h using T4 ligase (Takara).

Expression vectors were then transformed into *E. coli DH5α.* Clones were selected and identified using PCR and double enzyme digestion. Positive clones were confirmed by sequencing performed by Shanghai GeneCore BioTechnologies Co., Ltd., and were denoted EGFP-C1-p*Stra8*.

### 3.9. Cell Culture and Subcellular Localization

Pig SSCs were cultured in our lab from neonatal pig testis. The SSCs were isolated using the differential surface-attachment method [43,44]. The obtained cells were incubated for 4 h at 37 °C and 5% CO_2_. The culture solution was then pipetted gently and placed into another culture flask. After 3 h, the culture solution was pipetted again and the cells obtained in the culture media were purified as putative SSCs. Third generation SSCs and NIH/3T3 cells were transiently transfected to with *Stra8* to evaluate its subcellular location. Cells were cultured in Dulbecco’s Modified Eagle’s Medium (DMEM), 10% fetal bovine serum, 100 U/mL penicillin, and 100 g/mL streptomycin. All cells were grown at 37 °C, and 5% CO_2_. Lipofectamine™ 2000 reagent (Invitrogen) was used for transfection according to the manufacturer's instructions. After overnight incubation at 37 °C in 5% CO_2_, cells were imaged at room temperature using an inverted fluorescence microscope (Olympus, Shinjuku, Japan).

### 3.10. Statistical Analyses

All data are given as the mean ± standard error. Statistical analyses were performed using least significant difference (LSD) tests with SPSS version 13.0 (IBM Corporation, Armonk, NY, USA). A *p* value less than 0.05 was considered statistically significant.

## 4. Conclusions

In conclusion, this is the first report regarding pig *Stra8* and its spatio-temporal expression pattern. *Stra8* is thought to be meiotic gatekeeper gene, and its expression increases with age and bodyweight. Further studies are required to explain the function of *Stra8* as a potential transcription factor in meiosis initiation and to determine whether there are differences in phenotypes between various breeds of pig.
